# 279. Clinical Characteristics of Critically Ill Patients with COVID-19 and Invasive Pulmonary Aspergillosis: A Case Series From Mexico City

**DOI:** 10.1093/ofid/ofab466.481

**Published:** 2021-12-04

**Authors:** Patricia Galindo-Lopez, Benjamin Valente-Acosta, Francisco Moreno-Sanchez, Luis Espinosa-Aguilar, Irma Hoyo-Ulloa, Raquel Mendoza-Aguilar, Javier Garcia-Guerrero, Diego Ontañon-Zurita, Brenda Gomez-Gomez

**Affiliations:** 1 The American British Cowdray Medical Center, Mexico, Distrito Federal, Mexico; 2 The American British Cowdray Medical Center, Alvaro Obregon, Distrito Federal, Mexico

## Abstract

**Background:**

COVID-19 has emerged as a global public health emergency and has been the main cause of intensive care admission during the pandemic. COVID-19-associated pulmonary aspergillosis (CAPA) has been reported in case series of critically ill patients. However, the criteria for CAPA diagnosis has been inconsistent among most of the reports. Mexico has been widely affected by SARS-CoV-2. We present a series of CAPA cases at a teaching hospital in Mexico City.

**Methods:**

We performed a retrospective analysis of COVID-19 patients admitted to the ABC Medical Center from May 1^st^, 2020, to May 1^st^, 2021. Including only those with critical COVID-19 who required invasive mechanical ventilation (IMV). Patients with a diagnosis of CAPA were analyzed. We followed the 2020 ECMM/ISHAM consensus criteria for CAPA diagnosis. Aspergillus antigen testing in tracheal aspirate and serum was done with Aspergillus-specific galactomannoprotein (GP) ELISA (Euroimmun Medizinische Labordiagnostika).

**Results:**

Among the 230 admitted patients who required IMV, we identified 49 (21.3%) cases of CAPA, 46 probable CAPA and 3 proven CAPA. Nineteen (38%) of those died in the hospital. The mean age was 64.5 ± 12.6 years and 11 were female. Proven CAPA was diagnosed with culture in three cases (one *A. niger*, one *A. terreus* and one *A. fumigatus*). Probable CAPA was diagnosed by a positive serum GP in 27 (55.1%) patients and by a positive bronchoalveolar lavage (BAL) GP in 29 (59.2%) cases. Seven patients had both serum and BAL positive GP. Forty-six (93.9%) patients received corticosteroids, and 22 (49.9%) were treated with tocilizumab before CAPA diagnosis. All but one received isavuconazole as CAPA treatment. We detected 35 (71.4%) patients who had a bacterial co-infection. Eighteen of those died (51.4%) compared to only one dead in the subgroup without coinfections (7.1%). The mean time from hospital admission to CAPA diagnosis was 6.2 days (SD 7.1) among those who survived compared to 13.2 (SD 6.3) days in those who died p< 0.01.

**Conclusion:**

CAPA had a lower prevalence than previously reported in other series. However, it appears to be linked to high mortality when it occurs with other bacterial coinfections and when it is diagnosed late from admission.

**Disclosures:**

**All Authors**: No reported disclosures

